# KIR haplotypes are associated with late-onset type 1 diabetes in European–American families

**DOI:** 10.1038/gene.2015.44

**Published:** 2015-10-22

**Authors:** J A Traherne, W Jiang, A M Valdes, J A Hollenbach, J Jayaraman, J A Lane, C Johnson, J Trowsdale, J A Noble

**Affiliations:** 1 Division of Immunology, Department of Pathology, University of Cambridge, Cambridge, UK; 2 Cambridge Institute for Medical Research, University of Cambridge, Cambridge, UK; 3 Academic Rheumatology, University of Nottingham, City Hospital, Nottingham, UK; 4 Department of Neurology, University of California San Francisco School of Medicine, San Francisco, CA, USA; 5 Children's Hospital Oakland Research Institute, Oakland, California, USA

## Abstract

Classical human leukocyte antigens (HLA) genes confer the strongest, but not the only, genetic susceptibility to type 1 diabetes. Killer cell immunoglobulin-like receptors (KIR), on natural killer (NK) cells, bind ligands including class I HLA. We examined presence or absence, with copy number, of *KIR* loci in 1698 individuals, from 339 multiplex type 1 diabetes families, from the Human Biological Data Interchange, previously genotyped for HLA. Combining family data with *KIR* copy number information allowed assignment of haplotypes using identity by descent. This is the first disease study to use KIR copy number typing and unambiguously define haplotypes by gene transmission. *KIR* A1 haplotypes were positively associated with T1D in the subset of patients without the high T1D risk HLA genotype, DR3/DR4 (odds ratio=1.29, *P*=0.0096). The data point to a role for KIR in type 1 diabetes risk in late-onset patients. In the top quartile (age of onset>14), *KIR* A2 haplotype was overtransmitted (63.4%, odds ratio=1.73, *P*=0.024) and *KIR* B haplotypes were undertransmitted (41.1%, odds ratio=0.70, *P*=0.0052) to patients. The data suggest that inhibitory ‘A' haplotypes are predisposing and stimulatory ‘B' haplotypes confer protection in both DR3/DR4-negative and late-onset patient groups.

## Introduction

**Figure 1:**
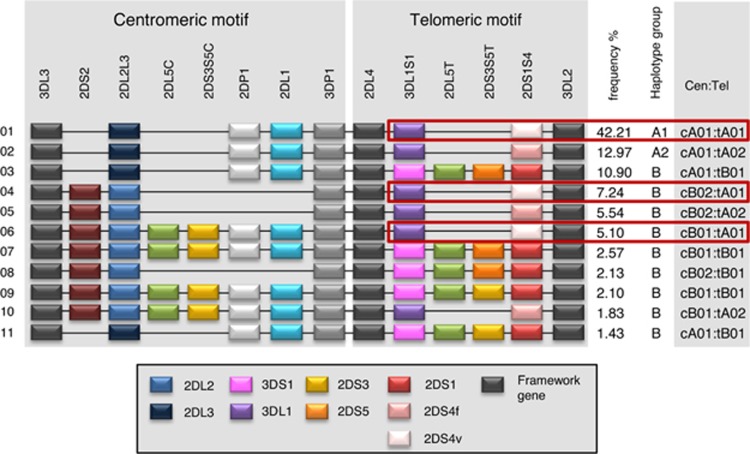
Schematic representation of common KIR haplotypes, with the predisposing tA01 gene group boxed in red. The gene content of tA01 and tA02 are the same, except that tA01 carries the 22 bp frameshift deletion form of *KIR2DS4* (2DS4v; 2DS4*003, *004, *006, *007, *008, *009,* 010, *012, *013), whereas tA02 encodes the full-length gene (2DS4f). Figure adapted from Jiang *et al.*^8^

Type 1 diabetes is an autoimmune disorder in which the insulin-producing cells of the pancreas are destroyed, resulting in a requirement for exogenous insulin for survival. Although >40 genetic loci are implicated in type 1 diabetes susceptibility, genes encoding the human leukocyte antigens (HLA) are the strongest genetic contributors.^1^ Killer cell immunoglobulin-like receptors (KIR) are found on the surface and regulate the function of Natural Killer (NK) cells, CD8+ and CD4+ αβ T cells as well as γδ T cells.^2^ Inhibitory KIR (KIR2DL and KIR3DL groups) have immunoreceptor tyrosine-based motifs within their long cytoplasmic tail. Activating KIR (KIR2DS and KIR3DS groups) have shorter cytoplasmic tails that are coupled via a charged residue in the transmembrane region to adaptor proteins, such as DAP12, which contain immunoreceptor tyrosine-based activating motifs. Ligands for KIR include class I HLA. HLA class I alleles are associated with type 1 diabetes, thus providing a biological rationale to investigate KIR association with type 1 diabetes.^3^ KIR haplotypes fall into two types: ‘A,' containing mostly inhibitory genes; and ‘B,' containing one or more activating KIR genes. Common haplotypes include centromeric (cen) and telomeric (tel) KIR gene groups, exhibiting strong linkage disequilibrium within, but not between, groups (Figure 1). The telomeric A (tA) group includes *KIR2DS4*, which is the only potentially short-tailed activating KIR on the A haplotype. However, *KIR2DS4* is disabled by a 22 bp frameshift deletion in exon 5 on the majority of Caucasian A haplotypes. This distinction defines tA01 (*KIR2DS4* full length) and tA02 (*KIR2DS4* deletion variant). A small number of studies have reported *KIR* association with type 1 diabetes; however, most are small case–control studies that focus on individual *KIR* genes and test only the presence or absence of *KIR* loci.^4^ A recent, larger study reports a method for imputation of *KIR* copy number for one *KIR* locus using GWAS SNP data but reports no significant type 1 diabetes association.^5^ In the current study, we performed *KIR* genotyping, with copy number determination, on a set of type 1 diabetes multiplex families that we previously genotyped for HLA class I and class II loci.^6^ This unique combination of family-based data with complete *HLA* and *KIR* copy number genotyping allows inference of individual haplotypes, based on identity by descent, for both *KIR* and *HLA*. We tested association of *KIR* haplotypes with type 1 diabetes in samples where full HLA context was known. Further, we tested those patients not carrying the highest-risk *HLA* genotype, that is, heterozygotes for DRB1*03:01-DQA1*05:01-DQB1*02:01 and DRB1*04:01/02/04/05/08-DQA1*03:01-DQB1*03:02 (referred to as ‘DR3/DR4') separately from the patients who do carry the high-risk genotype. Finally, given that HLA is associated with age of onset for type 1 diabetes,^7^ we examined the *KIR* genotyping data for association with age of disease onset.

## Results

All families in the Human Biological Data Interchange (HBDI) data set were complete, with two parents and at least two affected siblings. All individuals were genotyped for *KIR* presence or absence and copy number for each locus.^8^ All samples were previously genotyped for the loci *HLA-DRB1, -DQA1, -DQB1, -DPB1, −A, −B* and *–C* and exhibited Mendelian inheritance. Both *HLA* and *KIR* haplotypes were inferred using identity by descent. Common *KIR* haplotypes are illustrated schematically in Figure 1. Full haplotypes, containing both centromeric and telomeric portions, showed marginally significant overtransmission of *KIR* A1 haplotypes to affected individuals (*P*=0.0494). Separate analysis of centromeric and telomeric components of the *KIR* A1 haplotype (cA01 and tA01) revealed overtransmission of both to affected individuals, although neither result reached statistical significance. T1D patients were grouped into those who carry the very high-risk HLA class II genotype known as ‘DR3/4' (DRB1*03:01-DQA1*05:01-DQB1*02:01 and DRB1*04:01/02/04/05/08-DQA1*03:01-DQB1*03:02/04 or *02:01) and those who do not (‘non-3/4'). Transmission Disequilibrium Test (TDT) analyses revealed no significant transmission distortion in the high-risk DR3/4 group for any *KIR* haplotype tested. However, KIR A1-positive association with T1D was observed in the non-3/4 group, in which the HLA class II attributable risk is lower (*P*=0.0096, Table 1). Analysis of the data with Unphased 3.1.6 software also revealed this association (*P*=0.006, not shown). Separate analysis of telomeric and centromeric haplotypes showed significant overtransmission of the tA01 (*P*=0.0169, Table 1; Unphased: *P*=0.003, not shown). tA01 is included in ~55% of common *KIR* haplotypes in European populations (Figure 1). To test the hypothesis that KIR3DL1 may contribute to the T1D predisposing effect observed for tA01, the data were stratified by the presence or absence of HLA-Bw4, the ligand for KIR3DL1. Both the Bw4-positive and Bw4-negative subsets had overtransmission of tA01 (54.5% and 55.2%, odds ratio=1.23 and 1.20, respectively), with the Bw4-negative result reaching the threshold for statistical significance (*P*=0.05; data not shown). The fact that tA01 overtransmission is observed either in the presence or absence of the ligand for KIR3DL1 suggests that the KIR3DL1-Bw4 interaction is not the basis for the disease association of the tA01 haplotype.

HLA-C alleles can be divided into two groups, C1 and C2, depending on the identity of the amino-acid residues at position 77 and 80. *HLA* C2 alleles, which are ligands for *2DS1* and *2DL1*, are underrepresented in T1D, whereas C1 alleles, which are ligands for *2DL2, 2DL3* and *2DS2* (weak interaction), are overrepresented.^4^ Data from the current study were consistent with that report; C2 was undertransmitted (frequency transmitted =0.291, frequency not transmitted=0.336) to affected individuals, whereas C1 was overtransmitted (f-trans 0.709, f-not trans=0.66; *P*=0.000139) (data not shown). However, testing for gene–gene interaction between HLA-C allele groups and KIR loci showed no evidence of interaction for any KIR locus with C1 or C2 (data not shown).

*HLA* genes, particularly class I, are associated with age of onset for T1D, suggesting that *KIR* loci may also be associated in this manner. Mean age of onset for T1D patients was 11.22 years (range 0–36 years, s.d.=7.57; 95%CI=10.66–11.79); median age of onset was 10 years. Patients were grouped into four age of onset categories: (1) >0 and ⩽5 (*n*=181); (2) >5 and <10 (*n*=156); (3) ⩾10 and ⩽14 (*n*=197), (4) >14 (*n*=167). Analysis of patients in the top age of onset quartile (>14 years) revealed significant transmission distortion for several *KIR* haplotypes, including A2 (63.4%, p [TDT]=0.024; p[Unphased]=0.044) and B (41.1%, p [TDT]=0.0052; p[Unphased]=0.001), Table 2). No significant over- or undertransmission of *KIR* A and B haplotypes was observed in patients with age of onset 14 or younger. Notably, the *KIR* haplotypes that are associated in the over 14 age group (A2 and B, Table 2) differ from those that are associated in the non-DR3/4 group (A1, and more specifically, tA01, Table 1), although all data were consistent with overtransmission of *KIR A* haplotypes and undertransmission of *KIR B* haplotypes.

## Discussion

The fact that some HLA class I molecules are ligands for KIR leads to the notion that KIR may have a role in T1D susceptibility. Published reports of KIR association studies with T1D have little consistency among findings for particular loci (see allelefrequencies.net/diseases^4^ and references therein). Most are small case–control studies based only on presence or absence of individual *KIR* loci. The results reported in the current study are based on *KIR* haplotypes, inferred using identity by descent from family-based data, rather than on individual loci.

Our finding that the predisposing effect of the tA01 haplotype is more pronounced in the subset of T1D patients not carrying the high-risk DR3/DR4 genotype increases our confidence that the observed predisposing effect is real. This is consistent with previous studies of T1D risk loci other than HLA-DR- and DQ-encoding genes, including *TCF7*, *IL-4 R* and *PTPN22,* where the effect of the risk allele was stronger in the non-DR3/4 patients than in the DR3/4 group.

One report of functional studies in the NOD mouse model of T1D suggests that expression of the inhibitory receptor *KIR3DL1* predisposes NOD mice to diabetes by downregulating function of regulatory T cells.^9^ *KIR3DL1* is found on telomeric *KIR* A haplotypes; thus, our finding that tA01 haplotypes are predisposing for T1D is consistent with the NOD result. If the dominance of the inhibitory or educative activity of KIR3DL1 is important then presumably this should only be apparent when an appropriate HLA ligand (Bw4+) is also present. Although no statistically significant transmission distortion was observed in the HBDI data set for KIR in the context of HLA class I ligands, the combination of *KIR3DL1* with the gene for Bw4-80I (Bw4 molecules that have isoleucine at dimorphic position 80, which show stronger interaction with KIR3DL1 receptors than Bw4 molecules that have threonine this position (Bw4-80 T)^10, 11^) exhibited the highest transmission percentage at 54.7% (odds ratio=1.21, 95% CI 0.92–1.59). Using a combination of KIR copy number typing and SNP imputation of KIR copy number, Pontikos *et al.*^5^ report no association of 3DL1/3DS1 with T1D in a large case–control study; however, cases were not stratified by HLA, and age of onset for all subjects was less than 17. Although no firm conclusion about the risk effect of *KIR3DL1* on T1D can be made, the combination of evidence from genetic and animal models warrants further study of this locus. An alternative explanation for the tA01 association could relate to the presence of the *KIR2DS4v* gene that encodes a soluble and potentially secreted form of the protein. This may be biologically relevant if it antagonizes the effects of other KIRs.^12^

These haplotype-based data suggest that *KIR* genes have a role in T1D susceptibility. This study was powered with the assumption that KIR haplotype effects might be similar in magnitude to those of class I *HLA*; however, the effect of *KIR* diversity on T1D susceptibility appears to be modest. This does not rule out the possibility that individual *KIR* loci or haplotypes may have a substantial effect in a subset of patients. The data point to a role for KIR in type 1 diabetes risk in older onset patients, which has been missed in other studies. The fact that the A2 haplotype is more strongly associated with older age of onset than is the A1 haplotype suggests that the cell-surface receptor KIR2DS4 may have a role. However, in these data, patients who have at least one copy of *KIR2DS4* appear equally likely to have at least one ligand for that receptor (C*01:02, *02:02, *04:01, *05:01, *14:02 or *16:01) in the ⩽14 age of onset group compared with the >14 age of onset group (32% vs 33%, data not shown). A larger data set is needed to test each ligand independently. The data from this report support the notion that *KIR* A haplotypes, which contain mostly inhibitory KIR genes, are predisposing for T1D, whereas *KIR* B haplotypes, which contain more activating KIR loci, are protective. Activating KIR could act by stimulating regulatory T cells to control disease, or by influencing NK killing of effector T cells. On the other hand, inhibitory KIR could constrain the action of T1D-specific regulatory T cells, or fail to control cytotoxic T cells, allowing disease progression. In contrast with autoimmune diabetes in children, where HLA predominates, the genetics of autoimmune diabetes in adults are not well understood, despite its relatively high prevalence. Our finding will potentially be useful to better categorize patients in the future and to probe the influence of KIR on NK/T cells and autoantigen-specific regulatory T cells. Given the complexity of both the *HLA* and *KIR* genetic loci, and extreme variability of both among populations, a thorough understanding of the role of *KIR* genes and haplotypes in T1D will require very large sample sets, where individual subsets are large enough to uncover potential highly significant results.

## Subjects and methods

The HBDI is a repository for families including children with T1D (National Disease Research Interchange; http://ndriresource.org/Donor-Programs/Family-Genetics-HBDI/36/). These families were previously genotyped for the loci *HLA-DRB1, -DQA1, -DQB1, -DPB1, -A, -B* and *–C* and were analyzed extensively for T1D association.^6, 13^ The 339 families in this study are multiplex, that is, include more than one affected child. Parents are unaffected. All families exhibit Mendelian inheritance. HBDI families were genotyped for *HLA* class I and class II at two-field (four digit) resolution by polymerase chain reaction sequence-specific oligonucleotide probe (PCR-SSO) technology as previously described.^6, 13^ *KIR* genotype and copy number were measured using a quantitative PCR comparative Ct method developed from a protocol for determining genomic copy number as described earlier.^8^ Reactions were carried out in quadruplicate to ensure accuracy of the copy number scoring. Identity by descent-inferred KIR haplotypes were determined using the Merlin program as previously described.^8^ A TDT was applied to the data (two-sided test). Each family was analyzed as trios for each affected sibling. Odds ratios and 95% confidence intervals were estimated using the proportion T of transmitted risk allele as described.^14^ The estimate of the odds ratio is given by: T/(1−T). In addition, Unphased software 3.1.6^15^ was used to perform the association analyses using the allelemain model, which is robust to the issue of the non-independence of individuals in a sibling pair. The gxg model of the Unphased software was used to test gene–gene interaction between *HLA* allele groups and *KIR* loci. *P*-values are uncorrected based on prior hypothesis for KIR-T1D association.

## Figures and Tables

**Table 1:** TDT analysis of full, centromeric and telomeric *KIR* haplotypes for type 1 diabetes association with stratification by DR3/4 status

*KIR Haplotype*^a^	*Trans*	*N-trans*	*TDTtest*^b^	P*-value*^c^	*Trans %*	*OR (95% CI)*
*A B full, non-DR3/4*						
A1	236	183	6.70	**0.0096**	56.3%	1.29 (1.06–1.56)
A2	91	91	0.00	1.00	50.0%	1.00 (0.75–1.34)
B	283	314	1.61	0.20	47.4%	0.90 (0.77–1.06)
Others	37	59	5.04	**0.0247**	38.5%	0.63 (0.42–0.95)
						
*A B full, DR3/4*						
A1	128	121	0.20	0.66	51.4%	1.06 (0.83–1.36)
A2	53	53	0.00	1.00	50.0%	1.00 (0.68–1.46)
B	185	198	0.44	0.51	48.3%	0.93 (0.76–1.14)
Others	31	25	0.64	0.42	55.4%	1.24 (0.73–2.10)
						
*Cen haplotypes, non-DR3/4*						
cA01	421	381	2.00	0.16	52.5%	1.10 (0.96–1.27)
cB01	109	117	0.28	0.59	48.2%	0.93 (0.72–1.21)
cB02	80	90	0.59	0.44	47.1%	0.89 (0.66–1.20)
Others	37	59	5.04	**0.0247**	38.5%	0.63 (0.42–0.95)
						
*Cen haplotypes, DR3/4*						
cA01	232	220	0.32	0.57	51.3%	1.05 (0.88–1.27)
cB01	67	87	2.60	0.11	43.5%	0.77 (0.56–1.06)
cB02	67	65	0.03	0.86	50.8%	1.03 (0.73–1.45)
Others	31	25	0.64	0.42	55.4%	1.24 (0.73–2.10)
						
*Tel haplotypes, non-DR3/4*						
tA01	324	266	5.70	**0.0169**	54.9%	1.22 (1.04–1.43)
tA02	142	149	0.17	0.68	48.8%	0.95 (0.76–1.20)
tB01	144	173	2.65	0.10	45.4%	0.83 (0.67–1.04)
Others	37	59	5.04	**0.0247**	38.5%	0.63 (0.42–0.95)
						
*Tel haplotypes, DR3/4*						
tA01	181	189	0.17	0.68	48.9%	0.96 (0.78–1.17)
tA02	93	98	0.13	0.72	48.7%	0.95 (0.71–1.26)
tB01	92	85	0.28	0.60	52.0%	1.08 (0.81–1.45)
Others	31	25	0.64	0.42	55.4%	1.24 (0.73–2.10)

Abbreviations: CI, confidence interval; OR, odds ratio.

a A B Full=haplotypes containing both the centromeric and telomeric groups. A1=3DL3-2DL3-2DP1-2DL1-3DP1-2DL4-3DL1-2DS4Del-3DL2; A2=3DL3-2DL3-2DP1-2DL1-3DP1-2DL4-3DL1-2DS4FL-3DL2; B=haplotypes with at least one activating KIR gene; cA01=3DL3-2DL3-2DP1-2DL1-3DP1; cB01=3DL3-2DS2-2DL2-3DP1; cB02=3DL3-2DS2-2DL2-2DL5C-2DS3S5C-2DP1-2DL1-3DP1; tA01=2DL4-3DL1-2DS4Del-3DL2; tA02=2DL4-3DL1-2DS4FL-3DL2; tB01: 2DL4-3DS1-2DL5-2DS3S5T-2DS1-3DL2; others: haplotypes with frequency <1.0%.

b TDT tests were performed separately for each affected sibling in a family. Testing using an average of the two siblings for each family gave similar results; however, the *P*-values did not reach statistical significance.

c Significant *P*-values (> 0.05) are indicated with bold type. *P*-values are uncorrected based on prior hypothesis for KIR-T1D association.

Trans, number of transmitted haplotypes; N-trans, number of untransmitted haplotypes.

**Table 2:** TDT analysis of full, centromeric and telomeric *KIR* haplotypes for type 1 diabetes association with stratification by age of onset

*KIR Haplotype*^a^	*Trans*	*N-trans*	*TDTtest*^b^	P*-value*^c^	*Trans %*	*OR (95% CI)*
*A B full, age onset⩽14*						
A1	265	228	2.78	0.10	53.8%	1.16 (0.97–1.39)
A2	96	117	2.07	0.15	45.1%	0.82 (0.63–1.07)
B	362	361	0.00	0.97	50.1%	1.00 (0.87–1.16)
Others	45	62	2.70	0.10	42.1%	0.73 (0.49–1.07)
						
*A B full, age onset >14*						
A1	95	70	3.79	0.0516	57.6%	1.36 (1.00–1.85)
A2	45	26	5.08	**0.0241**	63.4%	1.73 (1.07–2.80)
B	102	146	7.81	**0.0052**	41.1%	0.70 (0.54–0.90)
Others	20	20	0.00	1.00	50.0%	1.00 (0.54–1.86)
						
*Cen haplotypes, age onset ⩽14*						
cA01	472	453	0.39	0.53	51.0%	1.04 (0.92–1.19)
cB01	139	147	0.22	0.64	48.6%	0.95 (0.75–1.19)
cB02	112	106	0.17	0.68	51.4%	1.06 (0.81–1.38)
Others	45	62	2.70	0.10	42.1%	0.73 (0.49–1.07)
						
*Cen haplotypes, age onset >14*						
cA01	173	140	3.48	0.0621	55.3%	1.24 (0.99–1.54)
cB01	37	56	3.88	**0.0488**	39.8%	0.66 (0.44–1.00)
cB02	32	46	2.51	0.11	41.0%	0.70 (0.44–1.09)
Others	20	20	1.00	1.00	50.0%	1.00 (0.54–1.86)
						
*Tel haplotypes, age onset ⩽14*						
tA01	375	333	2.49	0.11	53.0%	1.13 (0.97–1.31)
tA02	166	191	1.75	0.19	46.5%	0.87 (0.71–1.07)
tB01	182	182	0.00	1.00	50.0%	1.00 (0.81–1.23)
Others	45	62	2.70	0.10	42.1%	0.73 (0.49–1.07)
						
*Tel haplotypes, age onset >14*						
tA01	123	112	0.51	0.47	52.3%	1.10 (0.85–1.42)
tA02	66	55	1.00	0.32	54.5%	1.20 (0.84–1.72)
tB01	53	75	3.78	0.0518	41.4%	0.71 (0.50–1.00)
Others	20	20	0.00	1.00	50.0%	1.00 (0.54–1.86)

a A B Full=haplotypes containing both the centromeric and telomeric groups. A1=3DL3-2DL3-2DP1-2DL1-3DP1-2DL4-3DL1-2DS4Del-3DL2; A2=3DL3-2DL3-2DP1-2DL1-3DP1-2DL4-3DL1-2DS4FL-3DL2; B=haplotypes with at least one activating KIR gene; cA01=3DL3-2DL3-2DP1-2DL1-3DP1; cB01=3DL3-2DS2-2DL2-3DP1; cB02=3DL3-2DS2-2DL2-2DL5C-2DS3S5C-2DP1-2DL1-3DP1; tA01=2DL4-3DL1-2DS4Del-3DL2; tA02=2DL4-3DL1-2DS4FL-3DL2; tB01: 2DL4-3DS1-2DL5-2DS3S5T-2DS1-3DL2; Others: haplotypes with frequency <1.0%.

b TDT tests were performed separately for each affected sibling in a family. Testing using an average of the two siblings for each family gave similar results; however, the *P*-values did not reach statistical significance.

c Significant *P*-values (>0.05) are indicated with bold type. *P*-values are uncorrected based on prior hypothesis for KIR-T1D association.

Trans, number of transmitted haplotypes; N-trans, number of untransmitted haplotypes.
